# The Effects of Individualized Resistance Strength Programs on Knee Muscular Imbalances in Junior Elite Soccer Players

**DOI:** 10.1371/journal.pone.0144021

**Published:** 2015-12-02

**Authors:** Robert Śliwowski, Łukasz Jadczak, Rafał Hejna, Andrzej Wieczorek

**Affiliations:** 1 Department of Team Sports Games of the University School of Physical Education in Poznań, Poznań, Poland; 2 Rehasport Clinic FIFA Medical Centre of Excellence, Department of Physiotherapy, Poznań, Poland; University of Rome Foro Italico, ITALY

## Abstract

The purpose of this study was to investigate the effects of a resistance training program on the muscular strength of soccer players’ knees that initially presented unilateral and bilateral differences. For this study, a team of 24 male well-trained junior soccer players was divided into two strength program training groups: a Resistance Training Control Group (RTCG) composed of 10 players that did not have muscular imbalances and a Resistance Training Experimental Group (RTEG) composed of 14 players that had muscular imbalances. All players followed a resistance training program for six weeks, two times per week, during the transition period. The program of individualized strength training consisted of two parts. The first part, which was identical in terms of the choice of training loads, was intended for both training groups and contained two series of exercises including upper and lower body exercises. The second part of the program was intended only for RTEG and consisted of two additional series for the groups of muscles that had identified unilateral and bilateral differences. The applied program showed various directions in the isokinetic profile of changes. In the case of RTCG, the adaptations related mainly to the quadriceps muscle (the peak torque (PT) change for the dominant leg was statistically significant (p < 0.05)). There were statistically significant changes in RTEG (p < 0.05) related to PT for the hamstrings in both legs, which in turn resulted in an increase in the conventional hamstring/quadriceps ratio (H/Q). It is interesting that the statistically significant (p < 0.05) changes were noted only for the dominant leg. No statistically significant changes in bilateral differences (BD) were noted in either group. These results indicate that individualized resistance training programs could provide additional benefits to traditional strength training protocols to improve muscular imbalances in post-adolescent soccer players.

## Introduction

Soccer is an activity that requires intermittent explosive-type efforts, such as sprints, jumps, duels, and kicks, which depend on the efficiency of the neuromuscular system, particularly of the lower extremities [1]. All these activities need certain levels of strength and power from players in specific playing positions [2]. Most soccer players favor, or are forced to use, one particular leg for ball kicking and cutting skills [3–5]. This preference is the possible cause of asymmetry in the flexibility and strength of the lower extremities between the two legs, or between the agonist and antagonist muscles. Differences in strength profiles between the two legs are considered to be an important predictor of injury in soccer players [6–9]. Consequently, there is a need for investigations focusing on preventive interventions in soccer [10] and other sports involving asymmetric kinetic patterns, which are conducive to muscular imbalances.

Isokinetic testing of muscle strength is used for evaluating the effects of different kind of training programs for soccer players at various levels of training. Several programs have successfully incorporated one or more exercise components, including strength, strengthening neuromuscular control, static and dynamic stretching, plyometrics, balance, proprioception, running and cutting movement patterns, to prevent injuries in adult and youth soccer players [3, 4, 6, 10–19]. Some studies have demonstrated a significant reduction in the incidence and severity of musculo-ligamentous injury after appropriate resistance training [20–22]. A 10-week training program based on eccentric hamstring contraction was effective at developing maximal eccentric hamstring strength in well-trained soccer players, as demonstrated in two randomized studies concerning strength training [20, 22]. Arnason et al.’s [3] multi-year study confirmed these data. This study indicated that specific eccentric contraction training with Nordic hamstring lowers could reduce the incidence of hamstring strains in elite soccer players. On the other hand, Clark et al. [23] postulated that the combination of eccentric hamstring contraction training and traditional hamstring weight lifting movements might provide beneficial strength and length-tension adaptations to prevent soft tissue hamstring injuries. The question of whether the correction of any muscle imbalance could reduce the risk of injury has become a key problem for coaching and medical teams at various levels of soccer training.

Although it is evident that sport-specific strength training programs, including a balance training component, effectively improve physical fitness and reduce the risk of injury in adult athletes, there has been little research on such strategies in young and adolescent athletes [24]. The physiological adaptations to resistance training in youth athletes are largely related to the type of concentration, intensity and volume of load applied during the program. The average program in a meta-analysis on youth resistance training consisted of 2–3 sets of 8–15 repetitions with loads between 60% and 80% of the 1 repetition maximum (RM) on 6–8 exercises, however different combinations of sets and repetitions may be effective [25]. New perspectives for promoting resistance exercise as part of a long-term approach to youth physical development underline the significance of including resistance training in youth fitness programs [26]. Some studies have shown a positive effect of resistance training on motor performance and physical capacities in adolescents soccer players [27–29].

Resistance training-induced changes in limb muscular imbalances in adolescents are still a matter of debate. While this issue has been considered in relation to adult subjects, few studies have examined the influence of resistance training on the isokinetic peak torque of the hamstrings and quadriceps muscles, as well as the reciprocal balance of strength in the knee, in youth athletes. To our knowledge, there is only one study [30] related to young male soccer players. As such, there is insufficient scientific information regarding actual resistance training programs for adolescent well-trained soccer players. The improved strength of the hamstring muscles in the resistance-trained adolescents may have significant implications for the reduction of the risk and severity of musculo-ligamentous injuries [30]. Finding this type of relationship is extremely significant for researching youth professional soccer players.

Therefore, the purpose of the present study was to investigate the effects of a specific resistance training program on restoring the normal torque ratios of agonistic and antagonistic knee muscle groups in junior elite soccer players after the starting period. It was further hypothesized that resistance training program individualized in terms of muscular deficits will cause normalization of isokinetic parameters reducing any differences in this respect.

## Methods

### Design

The focus of new trends in rational training could be the risks associated with the imbalance and implementation of antagonist strengthening programs aimed at injury prevention [31]. The purpose of this study was to find potential imbalances in muscular strength in soccer players and to determine the effect of individualized resistance training programs on the reduction of bilateral differences and the recovery of normal torque ratios of agonistic and antagonistic muscle groups. The imbalance between the torque ratios of agonistic and antagonistic muscle group is referred to as muscular imbalance. Restoring the balance between agonist and antagonist muscle groups significantly decreases the risk of injury. For these reasons, a team of male well-trained junior soccer players was divided into two strength program training groups: those with and those without muscle imbalances. The basis for the division of the participants into the two groups was results from the isokinetic tests carried out at the beginning of the transition period (November 2012). The main training objective of this period was to increase the strength potential of the young soccer players, to reduce any differences in muscle imbalances, and a widely defined recovery after the starting period. Their resistance training program consisted of two parts. The first part, identical in terms of selected loads, was intended for both groups and covered all muscle groups. The second part was intended only for the players with muscle imbalances and aimed at reducing any differences in this respect. This training period served as a preparatory phase to prevent possible injuries from the high-intensity program, which would be applied during the preparatory period. The final tests (December 2012) verified the isokinetic strength profile of the studied players under the influence of the applied program of strength loads.

### Participants

Twenty-four elite junior male soccer players from a Poland First Division Club team took part in the study. They had been active in high-level soccer competitions (in the season when the study was carried out, the team won second place in the Polish U-17 championships). There were two groups of players: a Resistance Training Control Group (RTCG) composed of 10 players who did not have muscular imbalances (42%) and a Resistance Training Experimental Group (RTEG) composed of 14 players who had muscular imbalances (58%). Before the start of the study, the subjects were asked to complete a questionnaire to determine whether they had any musculoskeletal pain, discomfort or known injury in a lower extremity. Subjects were free of previous significant knee injuries and had no history of ACL repairs or rehabilitation. Each of the players and their parents or guardians were informed of the experimental risks and signed an informed consent document before the investigation. The participants were told that they were free to withdraw from the study at any time without penalty. Our study was conducted according to the Declaration of Helsinki, and the protocol was fully approved by the Human Research Ethics Committee before the commencement of the assessments. The basic physical characteristics of the players are summarized in Table 1.

**Table 1:** Physical characteristics and training experience of the subjects* (mean values ± SD).

	RTCG (n = 10)		RTEG (n = 14)	
	Pre-test	Post-test	Pre-test	Post-test
**Age (years)**	17.1±0.71	17.2±0.71	17.0±0.78	17.1±0.78
**Body height (cm)**	178.3±7.62	178.4±7.74	178.3±6.04	178.5±6.23
**Body weight (kg)**	71.6±7.56	71.9±7.58	69.4±7.32	69.7±7.21
**Training experience (years)**	7.3±2.32	7.4±2.32	7.1±2.53	7.2±2.53

* No significant changes in height or body mass between pre- and post-test in both groups (p<0.05).

### Procedures

#### Resistance Training Program

The study covered a 6-week training cycle, including the transition period of the 2012/2013 season. At the beginning of this period, the isokinetic strength profile of the players was determined, which was the basis for the division of the team into two groups. Each player was assigned an individual range of strength loads, and the training sessions with all of the methodological recommendations were organized.

Three familiarization sessions were performed at the beginning of the training program. Correct lifting techniques were explained and demonstrated to the players, who then practiced the exercises with light resistance. After this period, players began the strength training program, two times per week for six weeks, with a total of 12 training sessions. The strength training program was based on Bangsbo’s method [32] with small modifications. The program consisted of two parts. The first one, with identical selection of training loads, was intended for both training groups and consisted of two series of exercises. These series included a set of 12 upper and lower body exercises in the following order: seated legs press (a), standing back extension (b), standing leg adduction (c), standing arms abduction (d), seated flexion abdomen (e), seated single leg curl (f), standing leg abduction (g), seated oblique twist (h), seated single leg extension (i), flat bench press (j), climb on toes (k), and standing arms adduction (l) (all using machines with plates—STAR TRAC^®^, Irvine, CA, USA). Each of the series consisted of five repetitions at 80% of 1RM with 2–3 seconds between exercises and 3–5 minutes of rest between sets.

The second part of the training was applied only to RTEG players and consisted of two/three additional series of exercises (five to seven repetitions at 80% of 1RM—increasing progressively in the subsequent weeks) with 2–3 seconds between exercises and five minutes of rest between sets. Depending on the imbalance, the exercises in this part were performed for the muscle groups of a given extremity or extremities (exercises: a (single leg version)/, f/, i). Before the training session, each player received an individual work sheet with the recommended load range at each station. Each group trained separately (one after another) and the experimental part for RTEG was held at the end of the training, after a five minute rest. The specific configuration of the acute program variables during the course of the 6-week training period is presented in detail in Table 2.

**Table 2:** Training protocol of the resistance training program for both groups.

Week	Sessionsper week/no. of session	Exercise	Sets, repetitions, load(% of 1RM)and time betweenrepetition,(load for RTEG and RTCG)per session	Exercise(dependingon theimbalance)	Sets, repetitions, load(% of 1RM)and time between repetition,(load only for RTEG)per session
1	2/1–2	a−l*	2x5, 80% 1RM, (2–3 s)	a/ f/ i	2x5, 80% 1RM, (2–3 s)
2	2/3–4	a−l	2x5, 80% 1RM, (2–3 s)	a/ f/ i	2x5, 80% 1RM, (2–3 s)
3	2/5–6	a−l	2x5, 80% 1RM, (2–3 s)	a/ f/ i	2x6, 80% 1RM, (2–3 s)
4	2/7–8	a−l	2x5, 80% 5RM, (2–3 s)	a/ f/ i	2x6, 80% 1RM, (2–3 s)
5	2/9–10	a−l	2x5, 80% 5RM, (2–3 s)	a/ f/ i	2x7, 80% 1RM, (2–3s)
6	2/11–12	a−l	2x5, 80% 5RM, (2–3 s)	a/ f/ i	2x7, 80% 1RM, (2–3 s)

* type of exercise specified under Procedures

#### One Repetition Maximum (1RM) Testing Protocol

After the familiarization period, participants underwent the predicted one repetition maximum (1RM) testing for each of the exercises of the training program, except for the seated flexion abdomen and the seated oblique twist. Before the 1RM testing, all participants followed a standard warm-up routine consisting of one set of 10 repetitions with approximately 50% of their perceived submaximal loads using the correct movement pattern. To determine the submaximal strength loads, increments for each exercise were subsequently added until players failed to finish one repetition with proper technique. A 3- to 5-minute rest period was provided between each lift. Most subjects achieved their 1RM weight within four to five attempts. All measurements were performed with a constant position of the body, using the same resistance equipment by the same test administrator. The first author monitored the test session to ensure proper exercise technique and safety. To maintain an optimum training stimulus, individual 1RM training loads were readjusted at the end of three weeks of training for both groups.

The strength training session was the only training session in a day and lasted approximately 1.5 hours. A warm-up period of 10–15 minutes, including low to moderate intensity aerobic exercises and dynamic stretching, was carried out before strength training. A qualified strength training instructor, who monitored proper exercise techniques and made adjustments in training load and repetitions, supervised the strength training sessions. No injuries occurred during the training and testing sessions. Before the start of the study, the players, coaches and medical personnel were informed about the purpose and design of the study.

#### Soccer Training Program

The training program in the studied period (transition period) consisted of one 6-week training mesocycle. Microcycles (each week) were similar in structure and consisted of five days of training per week (Table 3). No games were played during the period in question. Strength training sessions took place on Mondays and Thursdays. Soccer training sessions took place on Tuesdays and Fridays, were of moderate intensity, usually lasted approximately 90 minutes and were mainly directed at the development of individual technical and tactical skills. Each training session generally consisted of a 15-minute warm-up, a 30-minute technical training, 40 minutes of small-sided games (4 vs. 4, 5 vs. 5), and a five-minute cool down. Dynamic stretching exercises for the main muscle groups were executed during the warm-up. Static stretching exercises were executed during the cool-down periods. Wednesday training sessions involved regeneration training and participating in additional sports or swimming.

**Table 3:** General activity content of weekly training.

Day of the week	Type of training
Monday	Strength training
Tuesday	Soccer training
Wednesday	Additional sports or swimming
Thursday	Strength
Friday	Soccer training
Saturday	Day off
Sunday	Day off

### Biodex’s Methodology Concentric Work

In this study, isokinetic knee strength was measured using the Biodex System 3 (Biodex Medical Systems Inc., New York, USA) dynamometer. The proper positioning and stabilization of the subject, the alignment between the axis of the rotation of the machine and the knee joint, the gravity compensation and the elimination of acceleration artefacts were performed according to the instruction manual for the Biodex Medical Systems and were similar to the ones described in the literature [14, 15, 30]. The warm-up that each player carried out before the isokinetic assessment took 10–15 minutes and consisted of mild pedaling on a stationary Monark cycle ergometer at a moderate pace (50–100 W) and dynamic stretches for the major lower-limb muscle groups [6, 8]. The concentric isokinetic torque of the quadriceps and hamstrings was assessed during continuous (bidirectional) knee extension-flexion movements at the angular velocity of 60° ·s^−1^ in three repetitions, through a knee range of motion of 0° (flexed) to 90° (full extension). Participants were given three trials at sub-maximal efforts with a gradually increasing load (50%, 75%, and approximately 100% of maximum capability) and then performed one set at the maximal concentric contraction. Subsequently, the same protocol was followed with the opposite leg. A 30-s rest was given after the third sub-maximal trial and a 3-min break was given when the machine setting was changed for the opposite leg. Standardized verbal encouragement was given before each maximal effort and visual feedback of the recorded torque was provided. The order of testing was randomized for the dominant (D) and non-dominant (N-D) legs [15, 23, 33]. Limb dominance was defined as the leg that is preferred when kicking a ball, and this was determined through an interview [6, 30]. The players were not subjected to a higher training load for two days before the measurements. All tests were conducted in the same order for each player during the pre- and post-tests, and all tests took place between 8 and 11 am [6, 8]. The same member of the research team performed all tests.

The resulting analysis included the absolute and relative peak torque (N·m and N·m·kg^−1^, respectively) for flexors (PT_Q_) and extensors (PT_H_) in both legs, unilateral ratio of muscle torque for both the dominant and non-dominant extremities (H_D_/Q_D_ and H_N_/Q_N_, respectively), and the bilateral ratio between the exerted strength of the knee extensors (Q_D_/Q_N_) and flexors (H_D_/H_N_). The net peak torque was used to calculate the bilateral leg strength differences (BD) and H/Q ratio. Bilateral leg strength differences were calculated by applying the Biodex System. In this study, the bilateral asymmetric of the knee muscles was defined as a strength imbalance of more than 10% [4, 8]. In the case of the H/Q ratio, a threshold of below 54.9% was defined as being deficient (this value was obtained by lowering the standard adopted by Biodex by 10%, i.e. 61%). The indicated limit adopted in our study, considered standard for this indicator, is basically in line with data suggested by Croisier et al. [10], who believed that values lower than 0.55 indicated a higher injury and re-injury risk.

The above norms were adopted in our laboratory in reference to teenage players. The division into two training groups was based on the data obtained for one velocity only. According to Houweling et al. [33], monitoring knee muscular asymmetries at a 60° ·s^−1^ velocity is the most valid indicator of previous injury to this at-risk muscle group in soccer players.

### Statistical Analysis

All analyses were performed using STATISTICA 10.0 (StatSoft, Inc., Tulsa, OK, USA). The results from the pre- and post-tests are reported as means with SD, while the changes within the RTCGs and RTEGs from the pre-test to the post-test are given as means with a 95% confidence interval. The distribution of the dependent variables was examined by the Shapiro-Wilk test. In order to test differences from the pre-test to the post-test for variables with normal distribution and homogenous variances, a paired t-test was used. For variables where one of the above conditions was not met, a non-parametric Wilcoxon’s test was used. Statistical significance was set at p ≤ 0.05.

## Results

The means and SD of the peak torque of the hamstring and quadriceps muscle groups are presented in Table 4. Changes of PT between the pre- and post-tests for the two training resistance groups varied. For the quadriceps muscle in the RTCG, statistically significant changes in absolute (220.5 ± 37.0 vs. 235.2 ± 37.1 N·m, p < 0.05) and relative (305.2 ± 29.1 vs. 326.6 ± 35.6 N·m·kg^−1^, p < 0.05) values of PT for the dominant leg were noted. No significant differences were noted in the case of the discussed indicator for the non-dominant leg. For the discussed group of muscles, no statistically significant differences of PT were noted in the RTEG. For the hamstring muscle in the RTEG, statistical changes were found related to the absolute (123.6 ± 20.1 vs. 136.2 ± 25.3 N·m, p < 0.05 –dominant leg; 118.9 ± 18.3 vs. 129.3 ± 15.9 N·m, *p* < 0.05 –non-dominant leg) and relative (177.1 ± 18.2 vs. 194.7 ± 23.2 N·m·kg^−1^, p < 0.05 –dominant leg; 171.1 ± 21.2 vs. 186.1 ± 16.8 N·m·kg^−1^, p < 0.05 –non-dominant leg) values of PT. Statistically significant differences were not noted for the discussed group of muscles for the RTCG.

**Table 4:** Isokinetic peak torque (PT) [values are mean (±SD)], and percentage of change (Δ) [values are mean (95% CI)] from pre- to post-tests.

	RTC Group (n = 10)				RTE Group (n = 14)			
	Pre-test	Post-test	95%CI	p Value	Pre-test	Post-test	95%CI	p Value
**PT**_**Q**_ **(Nm), D**	220.5±37.0	235.2 ±37.1	-23.51 to -5.91	0.004*	233.0±37.2	241.2±38.7	-18.37 to 1.95	0.104
**PT**_**Q**_ **(Nm·kg** ^**−1**^ **), D**	305.2±29.1	326.6 ±35.6	-34.81 to -8.02	0.005*	334.1±38.1	345.2±33.9	-25.64 to 3.39	0.121
**PT**_**Q**_ **(Nm), N–D**	222.6±33.3	230.9±28.8	-18.45 to 1.75	0.094	232.0±26.4	242.3±35.6	-23.38 to 2.82	0.113
**PT**_**Q**_ **(Nm·kg** ^**−1**^ **), N–D**	308.7±25.5	321.9 ±28.5	-28.31 to 1.75	0.076	333.8 ±25.8	347.5±31.7	-31.67 to 4.11	0.120
**PT**_**H**_ **(Nm), D**	132.1±21.4	134.8±25.2	-11.13 to 5.73	0.487	123.6±20.1	136.2±25.3*	-19.18 to -5.96	0.001*
**PT**_**H**_ **(Nm·kg** ^**−1**^ **), D**	183.5 ±17.0	187.2 ±26.3	-15.86 to 8.44	0.507	177.1±18.2	194.7±23.2*	-26.54 to -8.61	0.000*
**PT**_**H**_ **(Nm), N–D**	130.1 ±20.0	133.2±26.3	-13.47 to 7.31	0.519	118.9±18.3	129.3 ±15.9*	-15.12 to -5.61	0.000*
**PT**_**H**_ **(Nm·kg** ^**−1**^ **), N–D**	180.2±18.3	184.9±30.2	-19.09 to 9.67	0.477	171.1±21.2	186.1±16.8*	-21.72 to -8.19	0.000*

Q**—**quadriceps; H—hamstrings; D—dominant leg; N–D—non-dominant leg; CI—confidence interval; Nm = Newton metre;

* p < 0.05 significant differences between the pre- and post-tests.

**Fig 1:**
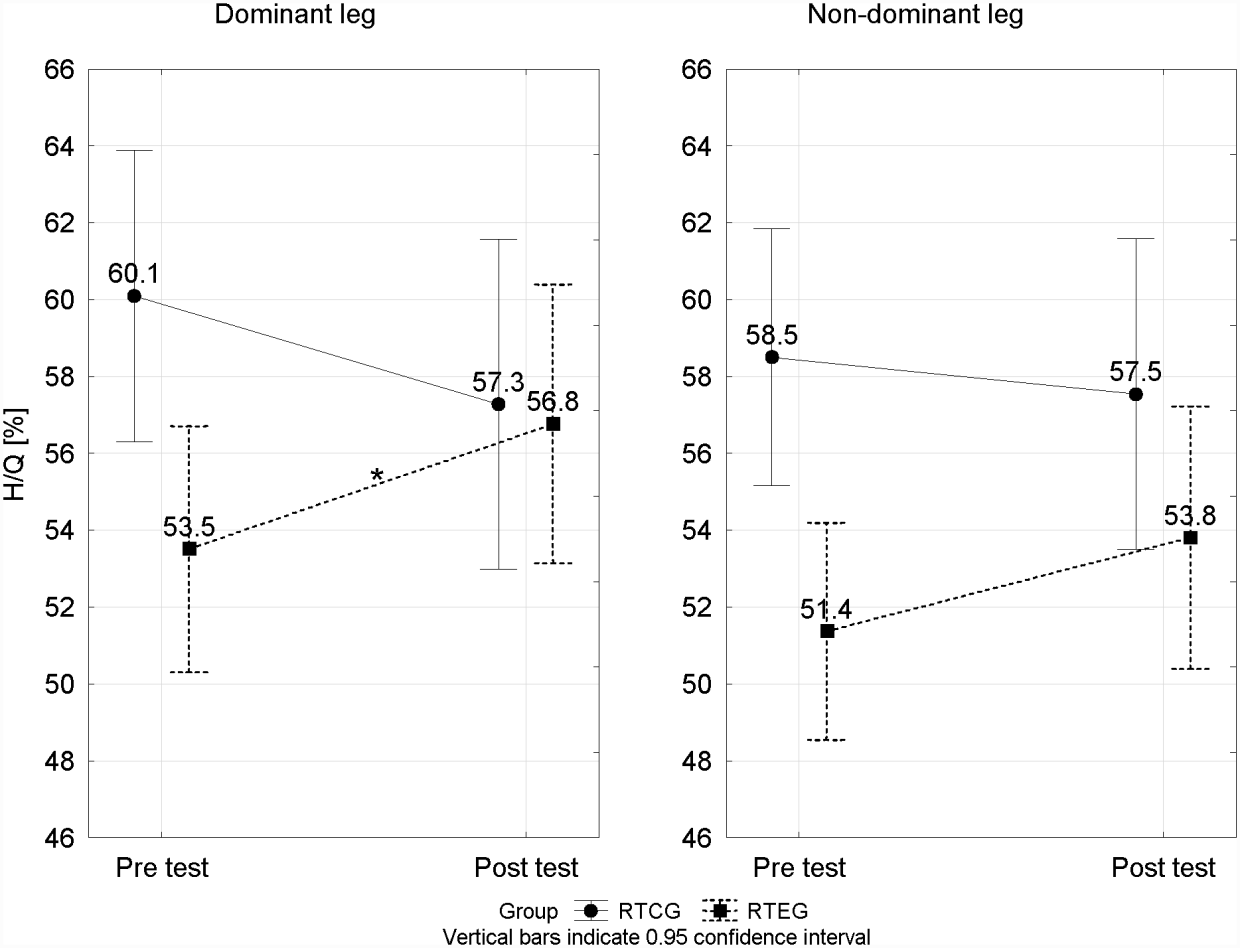
Comparison of changes in the H/Q ratio between the pre- and post-tests in two Training Resistance Groups. p < 0.05* significant differences from pre- to post-tests.

The analysis of changes in the H/Q peak torque ratio differences between the two training resistance groups provided some interesting data. The profile of these changes between the pre- and post-tests in the two training resistance groups is presented in Fig 1. In the case of the RTCG, the H/Q ratio changed from 60.1 ± 3.5 to 57.3 ± 4.3% for the dominant leg and decreased from 58.5 ± 3.2 to 57.5 ± 7.1% for the non-dominant leg. No significant differences were noted for the discussed indicator for any of the legs. A different direction of change was noted for the RTEG (Fig 1). For the dominant leg, the H/Q ratio changed to a statistically significant extent (p < 0.05), from 53.5 ± 6.9% to 56.8 ± 7.7%, and for the non-dominant leg, it increased from 51.4 ± 6.1% to 53.8 ± 5.4%.

**Fig 2:**
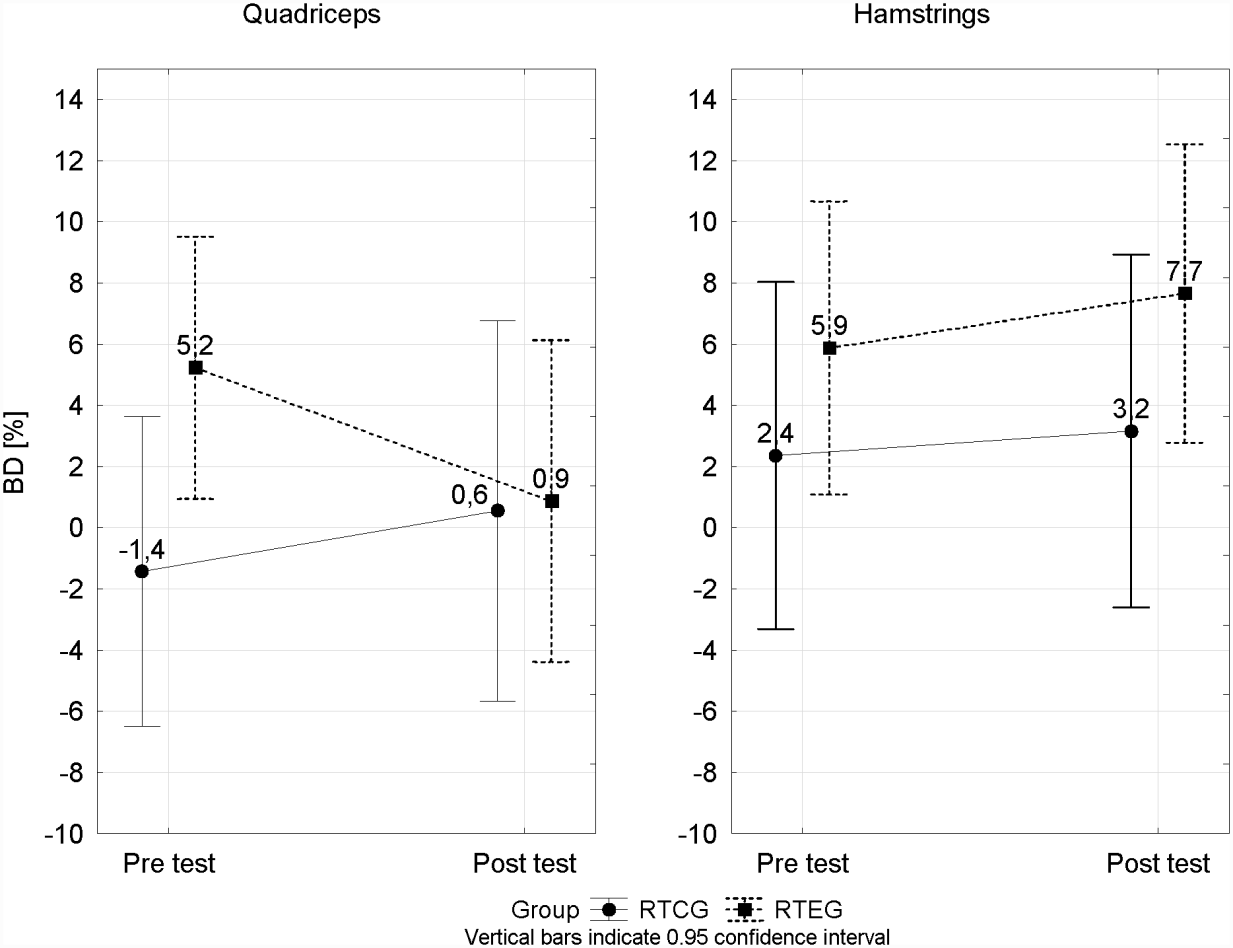
Comparison of changes in the BD between the pre- and post-tests in two Training Resistance Groups.

No significant changes were observed for the bilateral differences (BD) of knee extensors and flexors in any of the studied training groups (Fig 2).

## Discussion

Muscle injuries in soccer players may be effectively prevented with the implementation of screening examinations for muscle strength imbalances. In order to design an organized and controlled training process, it is essential to determine and quantify these imbalances in muscle strength, which would also serve as a tool to detect and compensate for them early in training. While these actions are regularly implemented in professional adult soccer players, a number of problems are encountered in training of youth players. These problems primarily result from the many limitations regarding access to the facilities and time, in addition to the lack of rational proportions between soccer-specific activities and functional or compensatory training. Many coaches often disregard the latter. With these limitations in mind, an attempt was made to optimize the training process of youth players during the transitional period, combining strength development with the reduction of muscle deficits within the knee joint.

The main result of this study showed that individualized training loads induced multidirectional changes in knee strength muscles within the studied training groups. In terms of the main isokinetic indication, which is PT, favorable changes were noted both for RTEG and for RTCG (Table 4). In the case of RTCG, the adaptations related mainly to the quadriceps muscle (for the dominant leg’s relative and absolute PT values, which were statistically significant (p < 0.05; Table 4)). These data might indicate that the program of training loads applied to RTCG primarily affected the strength of the quadriceps muscle. The improvement in the quadriceps’ strength was greater than in the hamstring’s strength, likely due to the nature of the running and kicking demands in soccer [6]. It seems clear that this muscle group is more often used, both in everyday life and in specific soccer training applications [1, 14, 21]. These data also confirm Daneshjoo et al.’s [13, 14] opinion that certain components of the intervention programs have a greater impact on the quadriceps’ isokinetic strength than on that of the hamstring. Hence, Iga et al.’s [30] noted that the muscle-loading patterns experienced by youth soccer players strengthen the muscles of the knee joint asymmetrically toward quadriceps dominance, compromising the capability for the hamstrings to provide muscular joint stabilization to the knee during high-velocity knee extensions.

With respect to RTEG, statistically significant changes (p < 0.05) are primarily related to the hamstring muscle. These results indicate that the main reason for the changes was an additional individualized program of loads applied mainly to this muscle group. It should be noted that in the case of RTEG, the deficits are related mainly to the flexors of the dominant and non-dominant legs. So in the majority of cases, the individualization of training loads was related to these muscle groups. These data also indicate that a lower level of use of hamstring muscles in everyday soccer training caused this muscle group to exhibit a higher reactivity to the additional set of exercises within a short period of time.

Our results agree with Golik-Peric et al.’s [34] results. These authors studied the effects of two specific training protocols on the isokinetic performance in a group of 38 adult athletes (including soccer players). The results in the RT group proved that resistance training induced almost double the level of hamstring strength changes than changes in the quadriceps. According to the researchers, this could be explained by the fact that initially the hamstrings were relatively weak, and the initial low value of concentric ratios supports this. Identical relationships in 15-year-old soccer players were confirmed in the study of Iga et al. [30] which demonstrated that the effect of 8-month training on hamstring muscles in resistance-trained groups with low (60° ·s^−1^) and high velocity (240° ·s^−1^) was higher (p < 0.05) compared with controls.

One of the studies with the largest number of participants in hamstring prevention research in adult soccer players [10] found that the risk of hamstring injury could be reduced thanks to detection and correction of isokinetic strength deficits. The studies from Scandinavian authors provide an insight into interesting relationships in the effect of resistance training on muscle injuries. The findings of the two studies on strength training referred to in the introduction indicated that a 10-week training program involving eccentric hamstring contraction activity effectively developed maximal eccentric hamstring strength in well-trained soccer players [20, 22]. Unfortunately, the Scandinavian findings were not verified in systematic studies on a larger group of players. In order to reduce the risk of injury, hamstring strengthening, particularly in the eccentric contraction mode, should be included in classical training routines [9, 12], because normalizing the isokinetic profile of soccer players that were found to have strength imbalances leads to a significant reduction in the frequency of hamstring injuries [10]. Moreover, the eccentric hamstring contraction training resulted in beneficial neuromuscular adaptations that could possibly prevent hamstring muscle injury in soccer players [23].

In soccer and other sports with asymmetric kinetic patterns, asymmetries in strength and flexibility between the two limbs and a reciprocal strength ratio between the agonist and antagonist muscles in the lower body play an important role [5, 8, 15]. It was suggested that strength imbalances, in particular in the lower body, might result in improper control of body movement [13, 15, 35]. Hence, the risk of injury is significantly decreased as a result of normalization of isokinetic parameters, by restoring strength balance between agonist and antagonist muscles groups around the knee joint [10]. The above issues suggest the main hypothesis of the present study that resistance exercises incorporated into the experimental training program may restore these muscular imbalances.

In the current study, the H/Q ratio changes, similar to peak torque, were of a multidirectional character between the two training resistance groups. In the case of the RTCG, a small decreasing trend was noted in the of H/Q ratio level in respect to both legs. All changes referred to above were statistically insignificant. The reason for a slight lowering of the H/Q level in this group was an increase in the strength of the quadriceps muscles, referred to above. A different direction of changes was noted for the RTEG. The H/Q ratio increased for dominant and non-dominant legs. Positive changes were found in as many as 86% and 71% of the studied players for the dominant leg and the non-dominant leg, respectively. This had an obvious relationship to the greater increase in the strength of hamstrings as compared to the quadriceps. The changes for the dominant leg were statistically significant (p < 0.05; Fig 1). It may be interesting that these changes related mainly to the dominant leg. Perhaps in this type of load, adaptation takes place sooner for the dominant leg. However, a confirmation of this hypothesis requires further study.

While our hypothesis was partially confirmed in relation to H/Q ratio, the results of the present study showed no significant bilateral differences in the knee extensors and flexors, for either of the studied resistance training program groups (Fig 2). There may be two reasons for the important limitations noted in this study. The first may relate to the applied training loads. The key factors in determining the outcome are training volume, intensity and progression, and the current exercise prescription seems to have been insufficient. Perhaps loads of insufficient intensity or duration could have contributed to this. The other reason might suggest isolating separate units of resistance training only for the given muscle groups of the weaker leg. A study carried out over a longer period could verify this type of reaction.

The study of Iga et al. [30] referred to above, indicates that the effects of training background in resistance-trained soccer players on the functional reciprocal muscle strength ratios for the knee flexion (H_CON_:Q_ECC_) and extension (H_ECC_:Q_CON_) at high velocity (240°· s^−1^) compared with conventionally trained soccer players were statistically significant (p < 0.05). No statistically significant intergroup differences were noted for the indicated training effect at slow velocity (60°· s^−1^). According to the authors these results suggest that the reciprocal balance of strength about the knee may be altered under high-velocity conditions by the muscle-loading patterns experienced in young soccer.

The study of Holcomb et al. [21] carried out on a group of 20-year-old female soccer players provided some interesting findings with respect to the effect of resistance training on H/Q ratios. These results suggest that 6 weeks of strength training that emphasizes hamstrings is sufficient to significantly increase the functional ratio (p < 0.05). No such relationships were noted for the conventional ratio. According to authors the type of training resulted in differential effect of training on the functional and conventional ratios. It should be noted that the indicated training effect was not limited to strength loads but included more functional training where agonist and antagonist muscles were acting together, while the functional ratio was affected to a greater degree.

The problems associated with the correction of any muscle imbalance in order to reduce the risk of injury, as well as whether muscle imbalance could cause injury, have not been thoroughly explored. In spite of many attempts at proposing prediction formulae to increase the accuracy of load determination in lower-body strength training exercises and the hamstring muscle activation proportion during these exercises [29, 36], optimal resistance training exercises for activating the hamstrings have not yet been established [36]. This indicates that further longitudinal studies are needed in order to investigate the effects of long-term involvement in soccer on the relationship between hamstring and quadriceps strength in the lower limbs at various levels of soccer training. We believe that a logically selected and targeted program of training loads between flexors and extensors in a regular resistance training program for junior soccer teams might be a successful method of injury prevention in terms of compensating for muscular deficits in the knee. This is confirmed by Iga et al.’s [30] earlier study, which suggests that including resistance training in exercise routines of youth soccer players can improve different knee muscle balances.

## Conclusion

This preliminary study showed that the individualized resistance training programs could provide various changes in the studied training groups. In a group of participants that were normalized in terms of knee muscular isokinetic strength, the applied loads affected primarily the quadriceps muscle. Introducing additional sets of exercises to the group with muscle imbalances resulted in additional benefits in the area of improvement of muscular deficits. These changes related mainly to the strength of the hamstring muscle, which in turn, resulted in an increase in H/Q. Interestingly, statistically significant changes related only to the dominant leg.

There are some limitations in the results of this study. The lack of fully-expected training effects, in particular with regard to BD, requires further observation in terms of rebuilding applied training loads. Therefore, future research should focus not only on the manipulation of training variables, such as intensity of volume, but also on appropriately-selected strategies to normalize imbalances that are separate for H/Q and BD.

Trainers and coaches in the process of strategic planning for training programs could apply the conclusions of the study to improving balance and strength of youth soccer players. In the context of our research, it seems that compensating for any muscle imbalances within the knee joint in well-trained junior soccer players should be a comprehensive and long-term process. Additionally, the proposed traditional individualized resistance training programs should be supported by negative (eccentric contraction) exercises as an element of the permanent training regime during the entire annual training cycle. This will not only contribute to the effective prevention of injuries, but also ensure the optimal development of the player’s functional potential, which will lead to a high sporting success in the senior category.
